# Implementing the free HPV vaccination for adolescent girls aged below 14 in Shenzhen, Guangdong Province of China: experience, challenges, and lessons

**DOI:** 10.1186/s40249-023-01149-1

**Published:** 2023-10-30

**Authors:** Dadong Wu, Peiyi Liu, Danhong Song, He Wang, Siqi Chen, Wanyi Tang, Xuelian Zhao, Fanghui Zhao, Yueyun Wang

**Affiliations:** 1 Affiliated Shenzhen Maternity & Child Healthcare Hospital, Southern Medical University, No. 2004, Hongli Road, Futian District, Shenzhen, 518000 China; 2 National Cancer Center/National Clinical Research Center for Cancer/Cancer Hospital, Chinese Academy of Medical Sciences and Peking Union Medical College, Beijing, 100021 China; 3 Institute of Hospital Management, Tsinghua University, Beijing, 100084 China

**Keywords:** Cervical cancer, Human papillomavirus, Vaccination, Adolescent, Case study

## Abstract

Cervical cancer is a major public health concern in China, accounting for almost one-fifth of the global incidence and mortality. The recently prequalified domestic bivalent human papillomavirus (HPV) vaccine offers a practical and feasible preventive measure. In response to the global call for action, the National Health Commission issued an *Action Plan* to eliminate cervical cancer by 2030, with promotion of the HPV vaccination for school-aged girls as a critical step. Despite this, implementation of the vaccination has been patchy, with very low coverage among eligible girls. To address this, from December 2021 to December 2022, a demonstration project was launched in Shenzhen, Guangdong Province, to promote the inclusion of HPV vaccine in local immunisation programme and to address existing barriers to implementation. Using multiple sources of data, this article presents a case study of the demonstration project, analysing its impact on rolling out HPV vaccination among eligible girls and identifying any challenges encountered during implementation. The demonstration project has shown promising results in increasing the HPV vaccination rate, promoting public awareness and acceptance of the domestic HPV vaccine, and establishing a model for quickly scaling up the vaccination at the municipal level. The success of the project can be attributed to several factors, including strong governmental commitment, sufficient funding, multi-sectoral collaboration, ensured vaccine accessibility and affordability, improved vaccination services, and effective health education and communication strategies. Lessons learned from Shenzhen can provide valuable insights for future advocacy and implementation of the vaccination in other areas of China, but challenges must be addressed to achieve universal coverage. These include addressing vaccine hesitancy, expanding the programme to cover a broader age range, and ensuring consistent quality of vaccination services in primary care facilities. Overcoming these challenges will require innovative strategies, public-private partnerships, and sustained funding and resources. Future research should focus on evaluating the long-term effectiveness of the vaccination programme and identifying contextual factors that may impact its implementation in different settings. Overall, the effective control of cervical cancer in China will rely on the “political will” to ensure the incorporation of preventive interventions into policies and universal programme coverage.

## Background

Cervical cancer is a major public health concern worldwide, with an estimated 604,000 new cases and 342,000 related deaths reported annually by the World Health Organization (WHO) [1]. China accounts for approximately 18% and 17% of the global incidence and mortality [2]. According to the National Central Cancer Registry, in 2016, the incidence and mortality rates of cervical cancer was 11.4 per 100,000 and 3.4 per 100,000, respectively [3]. Being characterised by an increasing trend, these rates pose a significant challenge to cervical cancer prevention in the country.

Persistent infection of high-risk human papillomavirus (HPV) is closely associated with cervical cancer, with over 95% of cases caused by the infection [4]. Scaling up HPV vaccination for girls aged 9–14 years, screening and treatment for pre-invasive and invasive cervical cancer are the key to reducing cervical cancer mortality and can contribute to achieving the Sustainable Development Goals [5]. In August 2020, WHO launched the first Global Strategy for Cervical Cancer Elimination [6], with a goal of vaccinating 90% of girls with an HPV vaccine by the age of 15 by 2030. In October 2021, WHO prequalified China’s first self-developed HPV vaccine, Cecolin™. This domestic vaccine is safe, highly efficacious against HPV 16 and HPV 18 [7], and more cost-effective than imported vaccines [8], making it a valuable addition to the global and national cervical cancer prevention efforts. In response to WHO’s call for action, in January 2023, the National Health Commission of China issued an *Action Plan to Accelerating the Elimination of Cervical Cancer (2023–2030)*, which aims to eliminate cervical cancer by 2030 [9]. The *Action Plan* sets stepwise targets for HPV vaccination, including promoting the vaccination of eligible girls through pilot programmes by 2025 and rolling out the programmes by 2030.

Since its initial adoption in Jungar Banner, Ordos, in 2020 [10], HPV vaccination pilots have been established in more than a dozen of cities in China, providing school-aged girls with either free vaccines or vaccination compensation [11]. For example, Ordos offered free imported bivalent vaccines to all girls aged 15–18 in the first year and extended the vaccination to newly turned 13-year-olds in the second year [10]. In Jinan, girls aged below 15 in Grade 7 are given free domestic bivalent vaccines [12]. Chengdu, on the other hand, offers subsidies of 600 Chinese yuan (CNY) per girl aged 13–15 for voluntary vaccination with domestic/imported bivalent or quadrivalent vaccines [13]. Both Jinan and Chengdu achieved vaccination rates of over 90% within 2 months in 2021 [14, 15], while Ordos reached a coverage of 70% by the end of 2022 [16]. Nonetheless, despite these pilot programmes, HPV vaccines are not yet part of the National Immunisation Programme (NIP), which significantly impacts the vaccination coverage [17]. The reality has been that less than 1% of Chinese girls aged 9–14 have been vaccinated with any HPV vaccine [8]. Furthermore, implementation of the pilot programmes can be affected by various factors, including shortages of HPV vaccines, cultural and literacy barriers, sensitivity to requesting a vaccine for a sexually transmitted infection, and negative events linked to vaccination that undermine public trust [18]. In addition, the programmes are mostly school-based, therefore can face challenges such as the belief that HPV vaccine is inappropriate for school-aged girls, offence of traditional norms, disagreement from schools, low priority compared to other health education issues, lack of governmental support, and hesitancy of parents and students [19].

Cervical cancer prevention scholars have suggested that conducting research on implementing HPV-based preventive strategies and advocating for their adoption by addressing the existing barriers could lead to significant benefits in reducing the burden of HPV-related diseases [20]. From December 2021 to December 2022, a demonstration project to include HPV vaccine in local immunisation programme was launched in Shenzhen, an economically developed city in Guangdong Province, Southern China. Funded by the Bill & Melinda Gates Foundation, this project aimed to promote the free HPV vaccination for eligible girls as a way to operationalise the cervical cancer elimination goal at the municipal level. Several key measures were adopted during the demonstration project, which seek to attract governmental attention, establish multi-sectoral collaboration, improve vaccine accessibility and affordability, enhance the capacity of primary care practitioners, and foster public awareness and acceptance of the vaccine and the vaccination programme. This article presents a case study of the demonstration project, analysing its impact on increasing the HPV vaccination rate among eligible girls and identifying any challenges encountered during implementation. Multiple sources of data were used, including policy-relevant documents, organisational reports, quantitative and qualitative survey findings, and data from the Shenzhen Vaccination Information System (SVIS). Through exploring the factors that contributed to the success or unsuccess of the vaccination programme, valuable lessons can be generated for future advocacy and implementation in other areas of China.

## Advocating for the free HPV vaccination for eligible girls

A more focused and sustained advocacy effort to address the challenges hindering uptake of HPV vaccination has the potential to significantly reduce the burden of cervical cancer [20]. In order to urge the municipal government to take action, the project team undertook a series of advocacy activities, including presenting international and national evidence on the burden of cervical cancer and effectiveness of HPV vaccination to policymakers, organising dialogs between key stakeholders (e.g., policymakers, health professionals, educators, and parents), and actively participating in the development of work guidelines. During these advocacies, HPV vaccination was framed as a key component of comprehensive cervical cancer prevention which can contribute to implementing the Healthy China Initiative [21], successfully stimulating policy attention. On December 20th, 2021, the project kick-off meeting was held between officials from the Shenzhen Municipal Health Commission, primary care practitioners, public health professionals, and academics. Prof. Youlin Qiao from the National Cancer Center, a recognised champion of China’s national initiative against cervical cancer, presented and led the discussion at the meeting, advocating for policy action. Subsequently, a mixed-methods baseline investigation was conducted, which comprised knowledge and attitude surveys of parents and primary care practitioners, as well as in-depth interviews with key stakeholders. The investigation proved useful in further attracting attention on HPV vaccination by raising awareness among multiple stakeholders, facilitating cross-sectoral cooperation (such as between the health commission and education bureau), and providing evidence for policy making and optimisation.

## Preparing for the free HPV vaccination

The policy advocacy described above was successful. On January 11th, 2022, the Shenzhen Municipal Health Commission, Education Bureau, Department of Finance, and Women’s Federation jointly issued the *Work Guidelines for Free HPV Vaccination for Eligible Girls (2022*–*2024)*, with the overall goal of vaccinating 90% of girls by the age of 15 by 2030. The *Work Guidelines* stipulated that, starting from September 2022, all girls entering Grade 7 and aged below 14 would receive free and voluntary HPV vaccination services, and two doses of the domestic bivalent vaccine must be completed before the girls reach age 15.

### Multi-sectoral collaboration

The *Work Guidelines* emphasised the importance of “governmental leadership, multi-sectoral collaboration, and social mobilisation” in achieving the goal, and established the responsibilities for all participating departments (Table 1). To ensure effective implementation, the Municipal/District Health Commission coordinated regular meetings and communication events among different departments to discuss and address challenges, share experiences, and streamline processes. The clear assignment of responsibilities and accountabilities helped strengthen programme implementation as all sectors were aware of their roles and held accountable for their actions. Some stakeholders perceived that the joint issuance of the *Work Guidelines* fostered a sense of unity and shared purpose in achieving the vaccination target. According to an official from a District Health Commission:

*“It’s a joint issuance, mainly from the Municipal Health Commission. They communicated with the Municipal Education Bureau. Once the document was released, the various districts were communicated with. They shouldn’t dare to neglect it, and the cooperation among various departments is very high.“ (A district health official)*.

**Table 1:** Accountabilities of departments involved in the free HPV vaccination for eligible girls in Shenzhen, Guangdong Province, China

Department	Accountability
Municipal/District Health Commission	Leading programme implementation Conducting programme monitoring and evaluation
Municipal/District Education Bureau	Registration of eligible girls and development of vaccination plans Collaboratively with Municipal/District Health Commission, conducting programme monitoring and evaluation
Schools	Data collection of eligible girls Organising inner-school health education and mobilising students, parents, and teachers Collaboratively with vaccination clinics, arranging eligible girls to receive the HPV vaccine
Municipal/District Department of Finance	Guaranteeing the vaccine procurement cost, vaccination service fee, health promotion and related project funds
Municipal Women’s Federation	Community and family health promotion
Municipal/District Maternity & Child Healthcare Hospital	Health education on cervical cancer prevention Programme management and evaluation
Municipal/District Center for Disease Control and Prevention	Collecting and assessing vaccine procurement plans, signing procurement contracts Distributing the vaccine Providing technical guidance for vaccination Monitoring and addressing suspected abnormal reactions to vaccination Managing programme data through the Shenzhen Vaccination Information System
Vaccination clinics	Managing vaccine use, vaccination registration, reporting data to district Centers for Disease Control and Prevention Health education and communication

*HPV* Human papillomavirus

### Centralised procurement with secured governmental funding

Different from other Category 2 vaccines in China [22], the domestic HPV vaccine is centrally procured at the provincial level of Guangdong. The Municipal Center for Disease Control and Prevention (CDC) of Shenzhen collects and assesses procurement plans from all 10 districts and reports to the provincial CDC by the end of March each year. After a unified bidding process at the provincial level, procurement contracts with suppliers are signed by the Municipal CDC. To ensure governmental funding, the free HPV vaccination for eligible girls was included as part of Shenzhen’s Livelihood Project, demonstrating a strong commitment from the municipal government. The Municipal and District Departments of Finance arranged subsidies of CNY 700 per eligible girl, including CNY 329 for the HPV vaccine and CNY 21 for the vaccination services each dose. The vaccine procurement cost is fully borne by the municipal finance, while the vaccination service fee is borne by the finance of the district where the vaccination clinic is located. Additionally, the municipal and district finances together provide CNY 30,000 and CNY 50,000 per year, respectively, to the Maternity & Child Healthcare (MCH) Hospital and the CDC for programme management. Through centralised bidding and procurement, the vaccine’s availability and accessibility are ensured. As an official from the Municipal Health Commission put it during the interview:

*“Funding is not an issue thus we are able to make such a commitment. The Livelihood Project is a strong commitment that the government makes to the people.” (A municipal health official)*

### Sound immunisation infrastructure

Shenzhen has a well-established vaccination system, with years of experience and a sound service infrastructure in place. Over the past years, the city has gradually improved its vaccine usage management and vaccination services, as well as the information system (i.e., SVIS) for vaccine circulation and administration. At community level, tight collaborations have been established between schools and primary care facilities, such as Community Health Service Centers (CHSCs) and district hospitals, which provide centralised vaccination services for school-aged children. These services include annual flu vaccinations and emergency vaccinations for diseases such as chickenpox. To facilitate effective coordination, communication channels, often utilising WeChat groups, have been established between schools and primary care facilities. In this partnership, school doctors play an active role in identifying eligible students and coordinating vaccination schedules with local vaccination clinics. They also actively engage with parents and guardians to obtain consent for vaccination and provide information about the importance of immunisation. This strong partnership has contributed to the smooth delivery of HPV vaccination services. As mentioned by a director of school-based vaccination:

*“If the vaccination is carried out in schools, one team consisting of one doctor and one nurse is sufficient. In the past, we used to vaccinate around 1000 students with the flu vaccine in one morning, with a team capacity of 200–250. Therefore, it is feasible for us to vaccinate an additional 100 girls with the HPV vaccine in one afternoon. Additionally, since we are able to efficiently transition between schools, it is possible for us to complete the vaccination in our community.“ (A director of school-based vaccination in a vaccination clinic)*

SVIS has been in use since 1997 and undergone several updates. The information system is known for its good logic validation performance, and both health workers and the public are proficient in using it. To facilitate implementation of the free HPV vaccination programme, a separate section has been added to SVIS, which simplifies the process of importing and using the vaccination data. This section clearly displays the vaccination status in different clinics and schools, providing crucial data for analysing vaccination situations and making arrangements for future work. According to a programme director:

*“We, as the management agency, have opened a dedicated query channel for the HPV vaccination programme on the information platform. This means that through the query channel, we can check the vaccination status of each school and primary care facility.” (A director of the HPV vaccination programme in a District CDC)*

### Public education campaigns

**Fig. 1:**
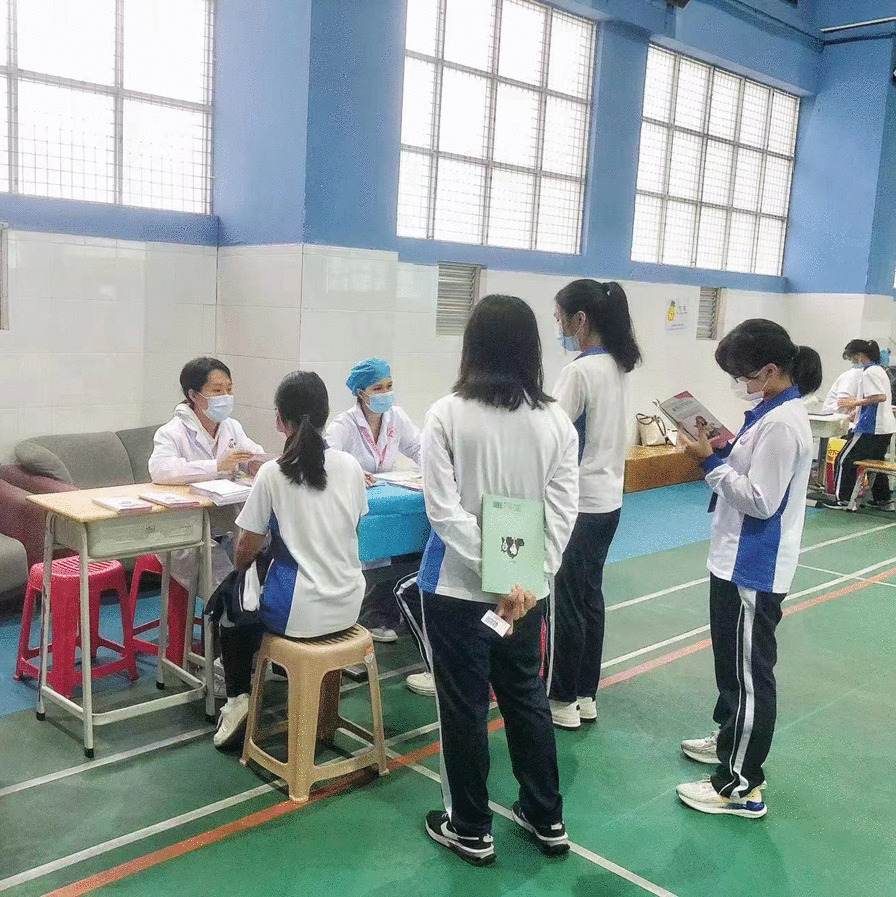
An “Expert-into-School” activity on HPV vaccination in secondary school. Source: Shenzhen Maternity & Child Healthcare Hospital, September 2022

During the promotion of HPV vaccination, health education played a pivotal role in raising awareness among the general public and in schools [23, 24]. The Municipal MCH Hospital and Women’s Federation conducted educational campaigns to inform the public about HPV, its link to cervical cancer, and the importance of HPV vaccination. Health education materials, including brochures and videos, were created and disseminated to District MCH hospitals and CHSCs throughout the city to be used in their routine health education efforts. To reach out to students and teachers, the MCH hospital launched the “Expert-into-School” programme (Fig. 1), where experts interacted with students and shared knowledge about HPV vaccination and cervical cancer prevention.

In addition, the city’s Adolescent Health and Development Programme has established connections with schools, which facilitates the integration of HPV vaccination into existing school-based health education programmes. As pointed out by a programme director:

*“For the HPV vaccine, we already have a project for adolescent health and development, so the cooperation between schools and the healthcare system is quite close. I just need to incorporate this into my adolescent health project, and we already have a platform and a foundation to work from.“ (A director of adolescent health education)*

Collaborating with the Municipal Education Bureau, the project team conducted a survey in June 2022 to assess the knowledge and acceptance of the HPV vaccine among parents of eligible girls, shortly before their daughter entered Grade 7. This survey was an important tool in promoting the vaccination and increasing adherence. The results showed that out of the 2856 parents surveyed, only 3.4% reported their daughters had already received any HPV vaccine. However, an overwhelming 97.3% of them expressed willingness to accept the HPV vaccination services provided by the government for their daughters, and 91.6% agreed that their daughters would receive the domestic bivalent HPV vaccine.

### Capacity building of primary care practitioners

In 2022, the project team organised five training sessions for primary care practitioners on cervical cancer prevention, which included interpretation of provincial and municipal policies, programme process, HPV vaccination, data input, quality control, cervical cancer screening, and mental health promotion for women who tested positive. Due to indoor gathering restrictions during the COVID-19 pandemic, all the training sessions were conducted online, reaching over 1000 trainees. At the end of each session, a questionnaire was distributed to assess the practitioners’ knowledge of and attitude towards HPV and HPV vaccine. This survey aimed to identify potential barriers to implementing the free HPV vaccination for eligible girls and collected baseline data for future evaluation of capacity building programmes. The findings, as published elsewhere [25], revealed that 94.7% of the primary care practitioners were willing to recommend HPV vaccination for their clients. However, those in private facilities had poorer knowledge compared to those in public facilities, such as CHSCs and district hospitals (Table 2).

**Table 2:** Knowledge of HPV and HPV vaccine among practitioners from various types of primary care facilities (*n* = 770). Source: Song et al. [25]

Type of facility	Average score	Score < 12	Score ≥ 12	Univariate logistic regression	
				*OR* (95% *CI*)	*P*
Level I CHSCs	12.14 ± 1.81	83	190	1.00	
Level II CHSCs	12.15 ± 2.15	92	216	1.03 (0.72–1.46)	0.889
Private facilities	10.89 ± 2.41	46	45	0.43 (0.26–0.69)	0.001
District hospitals	12.35 ± 1.94	32	66	0.90 (0.55–1.48)	0.680

*HPV* Human Papillomavirus; *CHSC* Community Health Service Center; *OR* Odds ratio; *CI* Confidence interval

## Implementing the free HPV vaccination

**Fig. 2:**
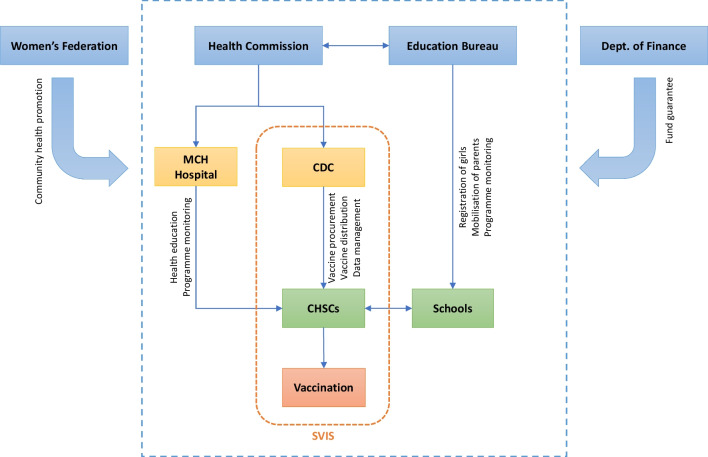
Implementation process of the free HPV vaccination in Shenzhen. MCH: Maternity & Child Healthcare; CDC: Center for Disease Control and Prevention; CHSC: Community Health Service Center; SVIS: Shenzhen Vaccination Information System

The free HPV vaccination officially began in October 2022, with a total of 60,524 eligible girls (aged below 14 in Grade 7 and never vaccinated for HPV before) registered according to data from the Education Bureau. The implementation process is illustrated in Fig. 2. As of December 14th, SVIS reported that 49,685 doses of free domestic bivalent HPV vaccine had been administrated, resulting in a free vaccination rate of 82.1%. With the addition of self-paid vaccines, it is estimated that the HPV vaccination rate among Grade 7 girls could reach 89% by the end of 2022, which is very close to the goal of 90% set by WHO as well as the municipal *Work Guidelines*. The rapid roll out of vaccination coverage acts as a testament to the successful implementation of the free HPV vaccination programme.

Nonetheless, certain limitations must be acknowledged. Centralised procurement of the vaccine can lead to supply chain and distribution challenges, potentially resulting in delays in the roll out of HPV vaccination. As noted by a programme director:

*“Initially, we anticipated a 60% coverage rate and procured 36,000 doses. When we realised this quantity was far from sufficient, we decided to administer the second dose as the first, as the second dose is administered six months later. We will procure more doses for the second dose as needed.“ (A director of the HPV vaccination programme in a District CDC)*

In addition, vaccine hesitancy among parents presents a significant barrier. Using an adapted 8-item Vaccine Hesitancy Scale (VHS) [26], the survey of parents of Grade 7 girls prior to the free vaccination found that, 2.1% of the parents had vaccine hesitancy (average VHS score ≥ 3). This proportion is lower than those reported in western countries [27, 28] but consistent with findings in other regions of China [29, 30]. As shown in Table 3, parents with vaccine hesitancy were more likely to reject the governmental HPV vaccination services (*χ*² = 153.309, *P* < 0.001). Moreover, among those unwilling to accept the vaccination services, 56.1% expressed a preference for imported or higher-valent vaccines, 10.9% were concerned about the vaccine’s safety and side effects, and 8.4% had little to no knowledge of HPV vaccine and HPV vaccination.

**Table 3:** Parents’ HPV vaccine hesitancy and willingness to accept the governmental vaccination services (*n* = 2856)

	Willing to accept (*n* = 2617)	Unwilling to accept (*n* = 239)
With vaccine hesitancy	28 (1.1%)	31 (13.0%)
Without vaccine hesitancy	2589 (98.9%)	208 (87.0%)

## Discussion

Cervical cancer is a highly preventable and treatable disease, yet it remains the world’s fourth most common gynaecologic cancer [1] and poses a significant burden in China, where an estimated 170 women die from cervical cancer every day [8]. The newly prequalified domestic bivalent HPV vaccine offers a practical and feasible preventive measure. It has been estimated that including the domestic vaccine in immunisation programmes is cost-effective both at the national level and in most provinces of China [31]. Nonetheless, despite the goal of vaccinating 90% of girls by the age of 15 by 2030, implementation of the HPV vaccination for eligible girls has been patchy across the country, with the overall vaccination coverage remaining extremely low.

Since 2020, pilot HPV vaccination programmes have been initiated in a number of cities, offering free HPV vaccines or vaccination subsidies for school-aged girls. The present case study highlights several critical factors contributing to the success of implementing the free HPV vaccination for eligible girls in Shenzhen, including strong governmental commitment backed with sufficient funding, active participation of multiple governmental departments, available and affordable vaccines, high-quality vaccination services, and improved public awareness. Comparing to implementation strategies adopted in many other parts of China, which mainly focused on provision of free vaccines and education to adolescents and their parents [11, 24, 32], Shenzhen introduced a holistic model that involved extensive policy advocacy activities, strong governmental leadership and clear accountability mechanisms, centralised procurement and streamlined distribution of the domestic vaccine, tight collaborations between schools and vaccination clinics, informatisation of vaccine administration, as well as targeted public educational campaigns.

However, when compared to cities like Jinan and Chengdu, where over 90% of eligible girls were vaccinated within 2 months [14, 15], Shenzhen’s free vaccination rate of 82.1% appears relatively lower. This discrepancy can be attributed to several factors. Firstly, in Jinan and Chengdu, HPV vaccination was extended to girls aged below 15 in Grade 7 and girls aged 13 to 14 at school, respectively. In contrast, Shenzhen’s *Work Guidelines* focused on vaccinating girls aged below 14 in Grade 7, potentially missing those who started school early. Moreover, centralised procurement of the HPV vaccine at the provincial level of Guangdong, as opposed to city-level procurement in Jinan and Chengdu, could have resulted in delays and logistical challenges in distributing the vaccine efficiently to vaccination clinics, impacting Shenzhen’s ability to rapidly reach a higher coverage.

### Lessons learned from the demonstration project in Shenzhen

While case studies in this domain remain limited, this in-depth analysis of Shenzhen’s experience offers a valuable reference point for understanding the intricacies of implementing the HPV vaccination for eligible girls at local level of China. Lessons learned from the demonstration project can provide valuable insights for future advocacy and implementation in other areas of China, particularly those have not yet initiated the vaccination. To begin with, given the rapid increase in the HPV vaccination rate among Grade 7 girls in Shenzhen after the municipal government began actively addressing it, it is imperative for local health agencies, professional societies, and non-governmental organisations to engage in more active, advocacy-oriented outreach to stimulate policy and funding attention. This aligns with findings from a policy analysis conducted in 2019, which suggested that the lack of powerful advocates and health professionals’ mobilisation skills hindered the feasibility of universal HPV vaccination in Shenzhen [33]. Advocacy efforts are more likely to succeed when advocates are tightly coalesced and able to transform international norms and up-to-date evidence into political influence, and link the issue to national political priorities, such as the Healthy China Initiative, thereby pressuring the local government to take action [34, 35]. The involvement of influential individuals, such as Prof. Youlin Qiao, and partnerships with networks focused on vaccine equity, like the Bill & Melinda Gates Foundation, present significant opportunities for effective advocacy [33, 36].

Additionally, strong governmental leadership and effective coordinating mechanisms are essential for achieving policy goals [35]. For the HPV vaccination for eligible girls, robust accountability frameworks should be established, building on existing immunisation and adolescent health infrastructures, to promote transparency, accountability, and collaboration at all levels. The demonstration project exemplified the effectiveness of clearly defined responsibilities for all participating sectors, which facilitated joint decision-making and collaborative implementation, particularly between CHSCs and schools, leading to the rapid expansion of vaccination coverage. This finding is consistent with existing literature that the most acceptable approach to improve vaccination uptake is through voluntary school-based vaccination programmes. For example, Lee and colleagues reported that a Home-School-Doctor model would improve adherence to HPV vaccination among adolescent girls and their parents even when they need to pay in Hong Kong, China [37].

Furthermore, Shenzhen’s experience underscores the importance of combining public educational initiatives with community outreach, such as the “Expert-into-School”, to enhance awareness and acceptance of HPV and HPV vaccine among parents, students, and school administrators. Targeted messages and tailored approaches effectively addressed specific concerns and misconceptions related to the domestic vaccine. Moreover, integrating health education on HPV into existing school-based sexual health curriculum through collaborations between schools and public health agencies proved instrumental in rapidly increasing positive attitudes towards the vaccination programme within a short time. This is consistent with experiences in other cities, as demonstrated in Zhang and colleagues’ multi-center intervention follow-up study, where a “Train-the-Trainer” approach significantly increased HPV-related knowledge and vaccine acceptability among adolescents [24].

### Challenges for implementing the HPV vaccination for eligible girls

The demonstration project also highlighted several challenges that must be addressed to further implement and sustain the progress. Firstly, vaccine hesitancy among parents and guardians due to concerns about the domestic vaccine’s safety and efficacy, coupled with limited knowledge about HPV vaccination, remains a significant obstacle. Notably, a recently published systematic review on HPV vaccination promotion interventions by Escoffery et al. emphasised the need to expand promotion efforts beyond educational interventions [38]. In this context, multi-component, multi-level and system interventions, targeting both individual behaviours and healthcare provider practices should be designed and evaluated [39].

Secondly, expanding the HPV vaccination programme to achieve universal coverage is a major challenge. Currently, the vaccine is only free for Grade 7 girls aged below 14, which leaves out many adolescent girls who are at risk of HPV infection. Moreover, girls who do not attend school are unable to access the vaccine, which limits the programme’s reach and effectiveness. To address these challenges, there is a need to develop innovative strategies to catch up with girls who missed the vaccine, especially those from vulnerable populations, and to expand the programme to cover a broader age range [40]. This could involve establishing public-private partnerships and a multiple funding mechanism, as well as engaging community health workers to improve vaccine access and uptake among hard-to-reach populations [41]. For example, a recently published randomised controlled trial by Li et al. reported that an innovative “Pay-it-Forward” approach, where participants received subsidised vaccines had the chance to donate to support others get vaccinated, significantly increased HPV vaccination uptake among girls aged 15–18, with a 98% uptake rate in the intervention group compared to 82% in the control group [42]. However, such efforts require a strong commitment from the government and stakeholders, as well as sustained funding and resources to ensure the sustainability and scalability of the programmes.

Thirdly, the applicability of Shenzhen’s experience to other areas of China is uncertain due to differences in economic development levels and healthcare systems. In an earlier health policy analysis, Chen et al. suggested that introduction of the HPV vaccination for eligible girls in Shenzhen was shaped by local legislative environment, economic development level, and social norms on immunisation and sexuality [33]. Therefore, it is important to conduct further research to understand the contextual factors that may impact implementation of the vaccination in different settings. Future research should also assess the long-term effectiveness of the vaccination programme, including its impact on the incidence and mortality of cervical cancer, and conduct health economic evaluations to support its expansion to other places. Given the dearth of case studies on implementing the HPV vaccination for eligible girls in China, this study not only contributes to address the research gap but also highlights the potential for applying the case study method in other cities. Cross-city case studies can enable comprehensive comparisons and enrich the knowledge base, ultimately facilitating the development of effective vaccination strategies.

Lastly, ensuring consistent quality of vaccination services in primary care facilities is crucial for the success of the HPV vaccination programme. Given that practitioners in private facilities had relatively poor knowledge of HPV and HPV vaccine, measures should be taken to ensure that they receive adequate training and resources to provide high-quality vaccination services. Future research should examine the drivers and obstacles to implementing the HPV vaccination for eligible girls at the primary care facility level, which will inform the design and testing of intervention strategies to optimise implementation. Such strategies could include targeted training and education programmes for practitioners, the establishment of quality assurance mechanisms, and incentivisation [38].

## Conclusions

Effective control of cervical cancer relies not only upon the availability of effective and affordable screenings and vaccines, but also on the “political will” to ensure that these interventions are incorporated into policies and that programme coverage is equitable and universal [43]. To achieve the policy targets over the long term, China’s national *Action Plan* for the elimination of cervical cancer must be followed by a series of ongoing, transparent, and operable policy initiatives combined with institutional and investment actions. Overall, the successful implementation of the free HPV vaccination for eligible girls in Shenzhen has demonstrated the significance of a comprehensive approach, combining effective policy advocacy, adequate financing, strong governmental leadership, multi-sectoral collaboration, secured vaccine accessibility and affordability, improved vaccination services, as well as public health mobilisation. Shenzhen’s experience underscores the critical role of voluntary school-based vaccination programmes in achieving the national and global goals of cervical cancer elimination and provides a valuable reference for other regions in China that are working to improve their cervical cancer prevention strategies.

## Data Availability

The survey and interview data collected for this study are not publicly available due to concerns about participant confidentiality. Access to the data may be requested from the corresponding author on reasonable grounds, subject to approval by the Medical Ethics Committee of the Affiliated Shenzhen Maternity & Child Healthcare Hospital, Southern Medical University.

## Abbreviations

WHO: World Health Organization
HPV: Human papillomavirus
NIP: National Immunisation Programme
SVIS: Shenzhen Vaccination Information System
CDC: Center for Disease Control and Prevention
CHSC: Community Health Service Center
MCH: Maternity & Child Healthcare
VHS: Vaccine Hesitancy Scale
OR: Odd ratio
CI: Confidence interval
